# HormonomicsDB: a novel workflow for the untargeted analysis of plant growth regulators and hormones

**DOI:** 10.12688/f1000research.124194.2

**Published:** 2024-04-08

**Authors:** Ryland T. Giebelhaus, Lauren A.E. Erland, Susan J. Murch

**Affiliations:** 1 Chemistry, University of British Columbia, Kelowna, British Columbia, V1V1V7, Canada; 2 Agriculture, University of the Fraser Valley, Chilliwack, British Columbia, V2R 0N3, Canada

**Keywords:** Plant metabolomics, phytohormones, synthetic biotransformations, hormonomics

## Abstract

**Background:**

Metabolomics is the simultaneous determination of all metabolites in a system. Despite significant advances in the field, compound identification remains a challenge. Prior knowledge of the compound classes of interest can improve metabolite identification. Hormones are a small signaling molecules, which function in coordination to direct all aspects of development, function and reproduction in living systems and which also pose challenges as environmental contaminants. Hormones are inherently present at low levels in tissues, stored in many forms and mobilized rapidly in response to a stimulus making them difficult to measure, identify and quantify.

**Methods:**

An in-depth literature review was performed for known hormones, their precursors, metabolites and conjugates in plants to generate the database and an RShiny App developed to enable web-based searches against the database. An accompanying liquid chromatography – mass spectrometry (LC-MS) protocol was developed with retention time prediction in Retip. A meta-analysis of 14 plant metabolomics studies was used for validation.

**Results:**

We developed HormonomicsDB, a tool which can be used to query an untargeted mass spectrometry (MS) dataset against a database of more than 200 known hormones, their precursors and metabolites. The protocol encompasses sample preparation, analysis, data processing and hormone annotation and is designed to minimize degradation of labile hormones. The plant system is used a model to illustrate the workflow and data acquisition and interpretation. Analytical conditions were standardized to a 30 min analysis time using a common solvent system to allow for easy transfer by a researcher with basic knowledge of MS. Incorporation of synthetic biotransformations enables prediction of novel metabolites.

**Conclusions:**

HormonomicsDB is suitable for use on any LC-MS based system with compatible column and buffer system, enables the characterization of the known hormonome across a diversity of samples, and hypothesis generation to reveal knew insights into hormone signaling networks.

## Introduction

With the growth of interest in metabolomics studies has come an explosion in the development of tools and databases for the annotation, analysis and interpretation of untargeted datasets. Fundamental to the ability to appropriately identify and annotate features in a dataset is the development of libraries and databases as the economics of purchasing standards to validate feature identification in these datasets is not feasible. While many tools and databases have been developed to assist in identification of metabolites from untargeted mass spectrometry (MS), much of the focus has been on capturing the largest possible depth and breadth of metabolites in a system. ^1^ While this increases the likelihood of matches often times identification using large databases, particularly for mass to charge (m/z) and retention time (RT) data or identification to level three confidence, ^2^ it also often leads to large numbers of improbable identities. This has led to calls for more specific databases, for example by sample type of metabolite class of interest which can improve the quality of matches and simplify the process. ^3^ The ability to search a dataset against a smaller, more focused database can therefore be helpful in identification of biologically relevant features within a dataset.

Hormonomics is a subset of metabolomics which has been defined as the study of the full spectrum of hormones, their conjugates and precursors in a living system. ^4^^,^^5^ The first hormonomics experiment is reported in Simura *et al.* 2018, where a targeted liquid chromatography tandem mass spectrometry (LC-MS/MS) method was developed to rapidly profile 101 phytohormones in plant tissues, including the bioactive forms of the hormones, their precursors, and their catabolites, allowing for a quantitative view of the state of the hormonome in the tissue being sampled. ^5^ This approach was then expanded and adapted to an untargeted metabolomics work flow. ^4^^,^^6^ Hormones are not however exclusive to plants and in fact are best studied in mammalian systems having been defined as “substance [s] which, being produced in any one part of the organism, is transferred to another part and there influence a specific physiological process”. ^7^ Hormones are often hard to quantify due to their labile nature and presence at low concentrations. Additionally, hormones, and phytohormones particularly are known to undergo diverse biotransformations, many of which remain uncharacterized, but which may play essential roles in their biological activity. ^4^^,^^8^ To avoid undesired induction of signaling cascade during storage or transport phytohormones are modified or conjugated to deactivate the compounds, while also allowing for the rapid release of these compounds when needed. To gain a full understanding of the hormonome of a sample all these possible forms must be considered.

We have developed a protocol using a standard buffer system, gradient and column which can be adopted for any LC-MS platform in coordination with the HormonomicsDB platform to specifically characterize the hormonome of a desired sample while also enabling discovery of novel phytohormone metabolites which may have physiological relevance. The inhouse hormonomics dataset described by Erland et al 2020a, b has been expanded and moved to a publicly accessible RShiny based web-tool “HormonomicsDB” ( http://hormonomicsDB.com) which allows for the putative identification of phytohormones, their precursors, metabolites, conjugates in untargeted datasets. We also performed a meta-analysis of data from 14 plant metabolomics studies archived on the Metabolomics Workbench using the HormonomicsDB web-tool to demonstrate the utility of the approach in exploring the plant hormonome. ^9^ Our previously developed synthetic biotransformations algorithms ^10^^,^^11^ are additionally integrated into the tool to allow for prediction and discovery of novel metabolites from MS metabolomics data using m/z and RT for compound ID.

## Methods

### Code, availability and issue reporting

**Figure 1.**
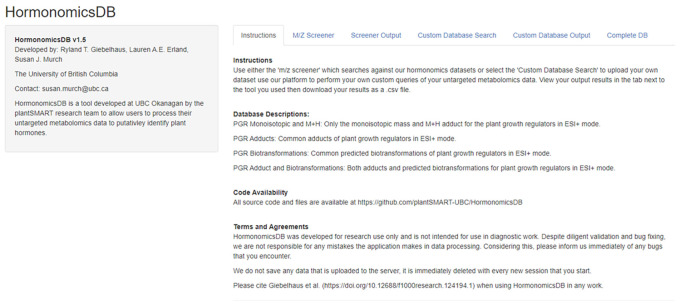
Screenshot of the current version of HormonomicsDB (v1.5) as viewed in Google Chrome.

The HormonomicsDB web-tool is available at http://hormonomicsdb.com/ ( Figure 1). The code was written using R ( https://www.r-project.org) and RStudio ( https://rstudio.com). To run the app, the RShiny package was utilized ( https://www.shiny.rstudio.com). The code of the current version 1.4 reported in this manuscript is archived on GitHub.com: https://github.com/plantSMART-UBC/HormonomicsDB.

### Plant growth regulators

A list of 249 plant phytohormones of interest was catalogued in a csv file, along with the class, monoisotopic mass, and M+H, m/z, InChI and SMILES terms. The list was expanded from those previously published in Erland *et al.* 2020 and Simura *et al.* 2018. ^4^^,^^5^ For each database entry, 7 common adducts and 27 synthetic biotransformations ( Table 1) are calculated. ^4^^,^^12^

**Table 1.** Description of the four searchable databases of plant growth regulators in HormonomicsDB.

Database Name	Database Description and Outputs
PGR Monoisotopic	Contains the monoisotopic mass of each archived plant growth regulator. Returns matches on the monoisotopic mass only.
PGR M+H	Contains the M+H adduct of each plant growth regulator and returns matches on this adduct only.
PGR Adducts	Contains and searches across 7 common adducts, M+H-2H _2_O, M+H-H _2_O, M+NH _4_-H _2_O, M+Li, M+NH _4_, M+CH _3_OH-H, and M+K, for all archived plant growth regulators.
PGR Biotransformations	Contains and searches across 27 common predicted biotransformations, M+CH _3_, M+C _6_H _12_O _6_, M+OH, M+COOH, M+NH _2_, M+NH _3_, M-H+OH, M-H+2OH, M-H+NH _2_, MCH _3_+H, M-CH _3_+OH, M-CH _3_+NH _2_, M-C _6_H _12_O _6_+H, MC _6_H _12_O _6_+OH, M-C _6_H _12_O _6_+NH _2_, M-C _6_H _12_O _6_+CH _3_, MOH+H _2_, M-OH+CH _3_, M-OH+C _6_H _12_O _6_, M-OH+COOH, MOH+NH _2_, M-OH+NH _3_, M-NH _2_+H _2_, M-NH _2_+CH _3_, MNH _2_+C _6_H _12_O _6_, M-NH _2_+COOH, and M-NH _2_+OH, for all archived plant growth regulators.

PGR Monoisotopic contains the monoisotopic mass for each phytohormone, the PGR M+H contains the m/z of each phytohormone as an M+H adduct, the PGR adducts contains the m/z of 7 common adducts ( Table 1), and PGR biotransformations contains 27 common biotransformations ( Table 1) as M+H adducts.

### Data input and output

User data is supplied via file upload as a comma separated value (CSV) file. A standard format is used for uploading data to HormonomicsDB, with m/z in the first column, RT in the second column, and sample intensities populating the remaining columns. Users can generate peak tables for upload to HormonomicsDB a number of ways, including with vendor software, or open source packages. These software take raw data files collected from individual chromatographic runs, and align the peaks to generate a single peak table which describes all the samples in the metabolomics experiment. These open source tools include XCMS (xcmsonline.scripps.edu), MetaboAnalyst ( metaboanalyst.com), mzMine (mzmine.github.io), and Metaboseek ( metaboseek.com). Given this, HormonomicsDB makes no assumptions about signal to noise (S/N) or other sensitivity figures of merit as these are typically made during this peak alignment step.

The maximum file size for HormonomicsDB has been set to 10 Mb for the web tool, however, this can be increased as desired when running locally in R by editing the source code manually. To demonstrate the features of the app, example data is provided on the app’s website, of which the details of the data can be found elsewhere. ^11^ Prior to data upload, the user selects the databases that they wish to search against; PGR Monoisotopic, PGR M+H, PGR Adducts, or PGR Biotransformations ( Table 1) and mass tolerance, which can be given as ± Da or ppm (Supporting Information ^37^). The user can then view the data in the “Screener Output” tab on the app or downloaded as a CSV onto the user’s computer by clicking the “Download Results” on the “m/z Screener” tab.

The databases queued against depend on the structure of the input data and the users hypothesis. Certain software, particularly for Fourier transform mass spectrometry (FT-MS) data, convert ion masses to monoisotopic mass before exporting a peak table. If this is the case, the user can queue against the “PGR Monoisotopic” database.

**HormonomicsDB custom search** The HormonomicsDB code was modified to create the “Custom Database Search” feature which allows users to use the existing search algorithm and user interface to search against a user uploaded database rather than the internal list of compounds. To use this feature, the user first makes their own database of compounds in a CSV using the same standard format as the “m/z screener”.

### Metabolite identification and the metabolomics standards initiative

HormonomicsDB putatively identifies metabolites on both m/z as well as RT. The query algorithm developed for HormonomicsDB first searches on m/z, selecting features that match to PGRs within the user specified search tolerance. Next these putatively identified PGRs are sorted on % RT match, calculating how close the experimental RT for the feature is to the predicted RT for the putatively identified PGR. HormonomicsDB putatively identifies metabolites to the metabolomics standards initiative (MSI) level 3, as two physicochemical properties, m/z and RT, are used to annotate compounds, without comparison to a chemical reference standard but which do not yield unambiguous matches. ^2^

### Standardized untargeted hormonomics protocol

An extraction protocol previously established for the extraction of phytohormones for LC-MS analysis was adapted for untargeted hormonomics. ^12^^–^^15^ For extraction, multiple different extraction solvents can be utilized to achieve the desired extraction. This includes water for polar extractions, as well as methanol for nonpolar extractions. Additionally, two extraction buffers used in previously published LC-MS protocols are recommended. The two extraction buffers are 0.5 N trichloroacetic acid (Fischer Scientific, Ottawa, ON, Canada) in 80% methanol (Optima, Fisher Scientific) and 50% methanol (MS Grade, Fisher Scientific, Canada; MeOH) and 4% acetic acid (glacial, Fisher Scientific, Canada) in Milli-Q water.

**Figure 2.**
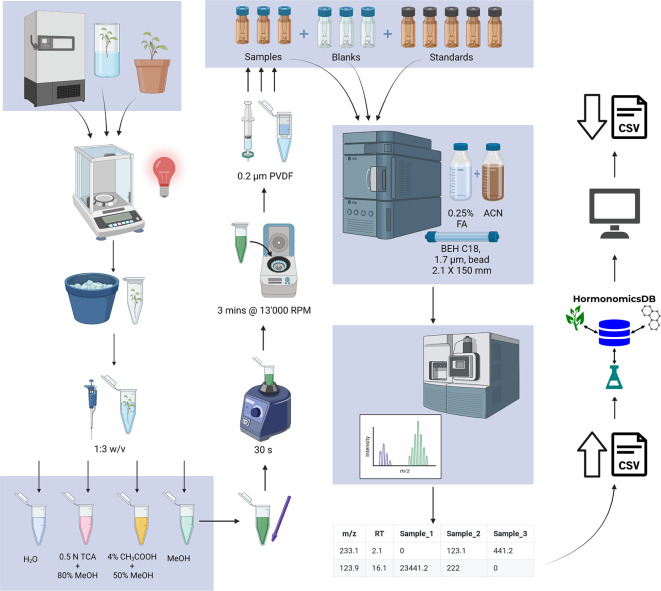
Standardized workflow for untargeted hormonomics analysis (Figure generated in BioRender).

All sample preparation is performed in a reduced light environment, under a red light. Approximately 100 mg of plant tissue was weighed into a 1.5 mL microcentrifuge tube ( Figure 2) and immediately stored on ice or in liquid nitrogen. Extraction buffer was added to the sample in a 3:1 volume (μL): mass (mg) ratio then homogenized on ice using a disposable tissue grinder (Kontes Pellet Pestle; Fisher). The resulting homogenate was vortexed for 30 seconds (Lab Dancer ©, VWR) then centrifuged for 3 minutes at 13,000 rpm (VWR Galaxy 16DH centrifuge). The supernatant was filtered using a 400 μL microcentrifuge filter (0.2 μm PVDF Ultrafree Centrifuge filter; Millipore, Etobicoke, ON, Canada) and centrifuged for 3 minutes at 13,000 rpm. If the sample matrix was complex, the filtrate could be further diluted with ultrapure water up to 1:10 filtrate: water. The filtrate was then aliquoted into an amber glass autosampler vial and stored at 4 °C prior to analysis.

An untargeted metabolomics method previously established in Brown *et al.* 2012 was developed with slight modifications. ^10^^,^^11^^,^^15^^,^^16^ Separation was optimized for use with Waters Acquity UPLC BEH C18 column (2.1 × 150 mm × 1.7 μm) at a temperature of 30 °C. A volume of 5 μL was injected onto the column at the start of the gradient. The elution was performed using eluents consisting of 0.25% formic acid (Sigma) in ultrapure water (Eluent A); 100% acetonitrile (Eluent B) was used with the following gradient: 0.0-10.0 min, 95:5-5:95 v/v, 10.0-15.0 min, 5:95 v/v, 15.0-20.0min, 5:95-95:5 v/v, 20.025.0min, 95:5 v/v. The needle wash solvent and purge solvent were both 10:90% water: acetonitrile (v/v). A pre-inject wash of 5 seconds was performed, followed by a 10 second post-injection wash. The total runtime was 25 minutes and a flow rate of 0.25 mL/min was used. The method should be compatible with most mass spectrometry platforms. For acquisition of MS data we have tested data acquire on a Waters LCT Premier to good success using positive electrospray ionization and positive ion detection, with capillary voltage of 2.9 kV, cone voltage 60 V, source temperature 120 °C, desolvation temperature 250 °C, mass range of 100–1000 amu and a scan time of 0.1 s.

### Retention time prediction

Using the validated method, a mixture of 46 known analytes were weighed then dissolved in the 0.5 N trichloroacetic acid and 80% methanol extraction buffer ( Table 2), each with a concentration of approximately 100 ng/mL were injected and eluted. Using the predicted M+H m/z for each analyte, extracted ion chromatograms were generated to determine the retention time of these analytes. These RTs were then catalogued in an Excel ^TM^ document.

**Table 2.** Metabolites used to build the retention time prediction model. All RTs and *∆* RT given in minutes.

Compound	Predicted RT	Experimental RT	*∆* RT
Histidine	2.45	1.23	1.22
Norepinepherine	3.57	1.35	2.22
Ascorbic Acid	2.74	1.85	0.89
5-Hydroxytryptophan	3.24	2.47	0.77
Zeatin	3.31	2.47	0.84
Phenylalanine	3.25	2.786	0.464
Indole	4.44	3.34	1.1
Caffeine	3.97	3.41	0.56
N-acetyl serotonin	5	3.704	1.296
4-chlorophenylalanine	3.99	3.81	0.18
Isopentenyl adenine	3.93	3.94	-0.01
Indole-3-acetic acid	4.2	3.94	0.26
6-Benzylaminopurine	4.53	4.07	0.46
Lidocaine	5.25	4.4	0.85
Coumaric acid	4.59	4.44	0.15
Gibberellin A3	5.63	4.492	1.138
Serotonin	4.52	4.51	0.01
5-chlorotryptamine	4.87	4.55	0.32
Vanillin	4.63	4.57	0.06
Strictosamide	4.85	4.621	0.229
Ferulic Acid	4.89	4.621	0.269
Anthranilic acid	4.58	4.69	-0.11
Melatonin	4.97	4.86	0.11
Kinetin	5.32	5.061	0.259
N-Feruloyl serotonin	4.67	5.079	-0.409
3-hydroxy tryptamine	5.64	5.281	0.359
Quercetin	4.92	5.3	-0.38
7-hydroxy mitragynine	5.41	5.648	-0.238
Mitragynine	5.73	5.65	0.08
Pipernal	6.04	5.85	0.19
Strychnine	4.67	5.9	-1.23
Reserpine	6	5.94	0.06
Indole-3-butyric acid	6.05	6.363	-0.313
Jasmonic acid	5.97	6.418	-0.448
1-Naphthaleneacetic acid	6.48	6.45	0.03
2-Naphthoxyacetic acid	6.43	6.8	-0.37
Ramelton	6.31	6.86	-0.55
Luzindole	6.87	6.86	0.01
Methyl Salicylate	6.9	7.17	-0.27
Melamine	6.49	7.68	-1.19
Brassinolide	5.91	7.76	-1.85
Paclobutrazol	7.16	7.89	-0.73
cis-4-Phenyl-2-propionamidotetralin (4-P-PDOT)	7.27	8.03	-0.76
Corticosterone	7.97	8.252	-0.282
Progesterone	6.95	8.33	-1.38
Nicotine	7.95	10.09	-2.14

Separation was performed according to the standardized protocol described above on a Waters Acquity I-Class ultra performance liquid chromatography system. Detection was on a Waters Xevo TQ-S in full scan mode (ESI+) using the following optimized settings: Mass range: 75.01100.0 m/z; capillary: 3.5 kV; cone voltage: 30.0 V; Source offset: 60.0 V; Source temperature: 150 °C; desolvation temperature: 400 °C; cone gas flow: 150 L/Hr; desolvation gas flow: 550 L/Hr; collision gas flow: 0.00 mL/min; nebulizer gas flow: 7.00 Bar. In addition to the injected samples, extraction solvent blanks and standards were injected between blocks of samples for quality control and to prevent carryover. In silico RT prediction was performed using the Retip package in the R environment. ^17^ The package used the in-house RT database as a training set and testing set for up to five ML methods; Random Forest (RF), bidirectional recurrent neural networks (BRNN), XGBoost, lightGBM, and Keras. Of these five models, two worked and were tested; RF and BRNN. The RF ML model was selected as the model to predict RTs for HormonomicsDB. Validation was performed by comparing the predicted RTs to experimental values of known compounds.

### Testing and validation

To test and validate the tool, a previously published untargeted metabolomics dataset generated using the same chromatographic parameters as described above was analyzed using HormonomicsDB. ^15^ The purpose of testing and validating was to assess the tools querying ability to ensure fit for purpose, while processing a large dataset. Additionally, we wanted to compare HormonomicDBs outputs to a previously published list of putative metabolites to determine if the tool returning the desired outputs.

This dataset was analyzed through HormonomicsDB using the ‘M+H’ database and a search tolerance of ± 0.02 Da. The putative hits from HormonomicsDB were then exported as a CSV, duplicate hits were removed, yielding a list of putatively identified metabolites. This list was then compared to the putatively identified analytes from Brown *et al.* 2012 to determine if ‘HormonomicsDB’ has any major bugs which cause issues in querying. The data in Brown *et al.* 2012 was collected on a time of flight (TOF) mass spectrometer, which typically has a mass error of 3 to 5 ppm.

### Meta-analysis of previous metabolomics studies

**Figure 3.**
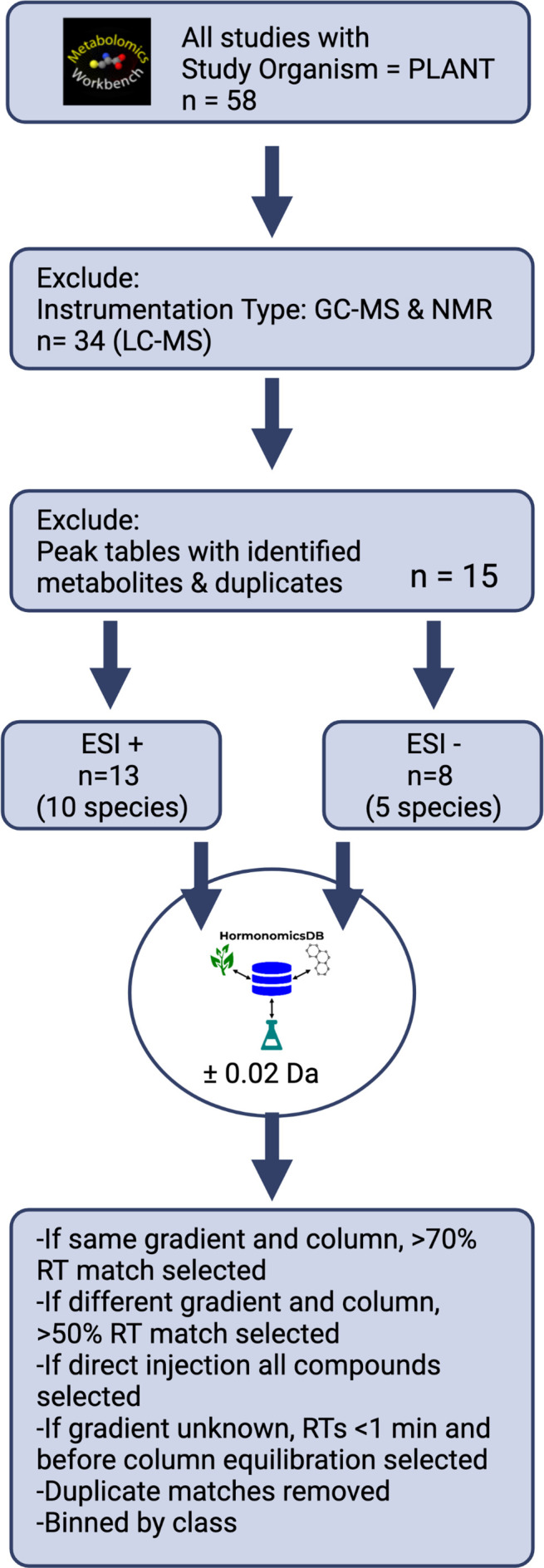
Overview of the steps in the meta-analysis of plant metabolomics studies from Metabolomics Workbench.

To demonstrate the utility of the HormonomicsDB webtool, we performed untargeted hormonomics analysis on all LC-MS the plant studies present on Metabolomics Workbench ( Figure 3). ^9^ First, studies with the study organism “PLANT” were selected, which as of June 2022 returned 58 studies. From these, studies which incorrectly returned a plant species, or used nuclear magnetic resonance (NMR), gas chromatography mass spectrometry (GC-MS), separation techniques such as hydrophilic layer interaction chromatography (HILIC), or reversed phase LC techniques with detection methods other than high resolution MS were excluded, leaving 34 studies. Of these, a number of studies had named peak tables, incomplete peak tables, or only raw, unaligned data. These were excluded further narrowing the number of studies to 14 ( Table 3). There were 13 studies, with 10 unique species, with peak tables acquired in ESI+ mode, and 8 studies with 5 unique species in ESI- mode. All these peak tables were formatted for HormonomicsDB and queued against the ‘M+H’ adduct with a mass tolerance of *±*0.02 Da. The output from HormonomicsDB was sorted by RT match. For studies with the same gradient and column used to construct the in silico RT prediction training set, compounds with an RT match >70% were retained. If the gradient and column were different compounds with a match >50% were retained. For studies where the gradient was not provided, RTs >1 minute and before column re-equilibration were retained. If direct injection was used, all compounds were selected. Duplicate compound hits were then removed and the compounds were binned by class.

**Table 3.** Summary of the 14 datasets archived on the Metabolomics Workbench explored in the meta-analysis of plant metabolomics studies.

Study ID	Study title	Species	Detection	Ionization mode
ST000240	Global LC-MS of Ozone Stress in Maize: GLCMS	Maize	OrbiTrap	ESI +/-
ST000289	PPDK RNAi effects in endosperm metabolite pools	Maize	OrbiTrap	ESI +/-
ST000290	2014 Biotron Experiment Metabolites	Maize	OrbiTrap	ESI +/-
ST000886	Mechanism Behind Stay Green Trait in Bread Wheat (Triticum aestivum L.)	Wheat	OrbiTrap	ESI +/-
ST001095	Data resource for fully 13C labelled and non-labelled plant tissues (part-I)	Arabidopsis thaliana	QTOF	ESI +/-
ST001096	Arabidopsis thaliana 25 accessions	Arabidopsis thaliana	QTOF	ESI +/-
ST001194	Flavonoid study of Ginkgo leaves facing to different elevation and plant age	Ginkgo biloba	TripleTOF	ESI -
ST001296	Metabolomics and Hormonomics to Crack the Code of Filbert Growth	Corylus americana	QTOF	ESI +
ST001330	Multi-omics of OsGF14b-mediated innate immunity against panicle blast in rice	Rice	OrbiTrap	ESI +/-
ST001371	Untargeted metabolomics of Quercus ilex acorns	Holly oak	QTOF	ESI +
ST001374	Untargeted Metabolomics for fruit juice authentication	Apple	TripleTOF	ESI +
ST001634	A combinatorial action of GmMYB176 and bZIP controls isoflavonoid biosynthesis in soybean.	Soybean	OrbiTrap	ESI +
ST001854	Metabolic profiling of Rafflesia-infected Tetrastigma and applications for propagation	Woody vine	QTOF	ESI +
ST002062	Endophytic bacteria are key players in the modulation of the secondary metabolome of Lithospermum officinale L.	Common gromwell	OrbiTrap	ESI +

## Results and discussion

### HormonomicsDB web-tool functionality and interface

The HormonomicsDB web-tool is an open source web tool developed using RShiny allowing users to perform compound annotation of untargeted metabolomics datasets using retention time and accurate mass matching ( Figure 1). The database includes 249 hormones which were originally assembled from the plant science perspective. In addition to matching to monoisotopic mass the database allows for matching to common adducts as well as predicted metabolites through the synthetic biotransformations approach ( Figure 2; Table 1). Data is uploaded in.csv format and results can be viewed either on the web or downloaded as a .csv file. The search functionality may also be used to search any two datasets given they contain m/z.RT data.

By comparing the putatively identified phytohormones from Brown *et al.* 2012 to the output of HormonomicsDB we determined the tool was fit for purpose. ^15^ The match rate was 104.5%, and was considered acceptable as our algorithm matches first on m/z then on RT, therefore, two or more isobaric phytohormones may return for a given m/z ( Table 4).

**Table 4.** Top 10 synthetic biotransformations by total signal intensity as output from HormonomicsDB found in the dataset published by Brown *et al.* 2012. ^15^

Compound Name	Biotransformation	Actual m/z	Experimental m/z	RT (min)
5-(3’-carboxy-3’-oxopropyl)-4,6-dihydroxypicolinate	M-NH _2_+C _6_H _12_O _6_	419.08	419.10	2.68
glucobrassicin	M+NH _3_	465.09	465.10	3.50
IAA aspartate	M-C _6_H _12_O _6_+OH	287.07	287.05	2.63
7,8-dihydroxykynurenate	M-NH _2_+C _6_H _12_O _6_	385.08	385.06	1.38
2-methylthio-cis-zeatin riboside	M+C _6_H _12_O _6_	577.21	577.20	3.45
IAA glutamate	M-C _6_H _12_O _6_+OH	301.08	301.70	2.92
4,6-dihydroxyquinoline	M+C _6_H _12_O _6_	341.11	341.10	3.62
4,6-dihydroxyquinoline	M-CH _3_+H	147.03	147.04	3.61
4,6-dihydroxyquinoline	M-NH _2_+H _2_	147.04	147.04	3.61
IAA glutamate	M-C _6_H _12_O _6_+OH	301.083	301.07	2.77

Additionally, the “HormonomicsDB custom search” function is a novel feature within HormonomicsDB which allows users to search their peak table against their own database of metabolites. This is the first such report of a feature that allows users to queue with their own database. We hope to continue growing this function to allow users to queue against other databases by accessing them through an application programming interface (API) in the HormonomicsDB environment.

### Development of the standardized protocol

In order to facilitate high confidence compound identification, a standardized and easily adapted LC-MS method was developed that accounts for the low concentration, labile nature of hormones and also accounts for the differential polarity of some phytohormones ( Figure 2). The method is derived from established protocols and uses equipment and solvents already present in most biology, chemistry and analytical labs and is outlined in Figure 2. ^13^^,^^18^^,^^14^ Special consideration was applied to extraction conditions as phytohormones represent unique challenges as analytes. First, in sample preparation, keeping samples on ice in a low light environment helps to prevent the degradation of labile phytohormones such as melatonin and auxin. ^13^^,^^19^^,^^20^ Typically, degradation of analytes during sample preparation is negligible for quantitative work, however, hormones often occur at levels approaches detection limits, particularly in untargeted studies making extraction losses a significant concern. Another concern is the potential for reactions during the extraction process which may lead to modification of structures and potentially loss of the biologically relevant forms or bias in the forms observed in the dataset. The use of methanol and water in the extraction solution encourages the extraction of both polar and non-polar analytes. Acidification of the extraction buffer with acetic acid or trichloroacetic (TCA) acid helps to acidify the solution to increase the solubility of phytohormones and protonation increase ionization efficiency in ESI+. TCA also precipitates proteins, and provides ion paring to reduce ion suppression in the mass analyzer. ^21^

### Database curation

The databased includes 249 metabolites which are established hormones or precursors or metabolites thereof. Classes of hormones include both naturally occurring and synthetic hormones with broad class coverage of: catecholamines, indolamines, auxins, jasmonates, salicylates, cytokinins, gibberellins, polyamines, butenolids (karrikins and strigolactones), steroids and abscisic acid. While development of the initial database has been from a plant hormone perspective, it also represents good coverage of the overall hormone landscape across Kingdoms. Only known metabolites have been included in the database, though the capacity to predict novel metabolites is built into the predictive biotransformations functionality. Inclusion of molecular formula, SMILES and InChI terms facilitates interoperability between database search results and classification tools such as MetaboAnalyst, ChemRich or ClassyFire. Accurate mass and molecular formula are available for all database entries, predicted retention time is also included for all entries and was predicted for the standardized method using the Retip App ^17^ to allow for compound identification to MSI Level 3 as the presence of isobars within the database leads to the possibility for non-unique matches even with integration of retention time. ^2^ The complete database is available in .csv format in Supplementary File 1.

### Retention time prediction

The advent of machine learning algorithms has led to several important tools which facilitate compound identification. We have applied the ReTip app to allow for retention time prediction within the database, given users have uploaded data generated from the standard separation protocol or one similar. The random forest model for retention time prediction showed the best fit to the training data with an R2 = 0.92 ( Table 5). Both the standard error (1.1 min) and the 95% confidence interval (± 1.17 min) were lowest of the models tested and were considered acceptable for the purpose. Manual plotting of predicted vs experimental retention time showed a highly linear relationship with R ^2^ > 0.9 and slope of 1.39. We anticipate that the predictions will be robust for LC-MS based systems running the standard separation protocol. While RT predictions help to increase confidence in putative identification, it is important to note that differences in gradient, column, or solvents can impact the RT of analytes. The users should place more emphasis on the accurate mass matching and use RT prediction results to confirm if the elution pattern or order is as expected and elution of the analyte occurs at a reasonable time within the gradient.

**Table 5.** Accuracy of machine learning models used for retention time prediction. BRNN; Bidirectional recurrent neural networks. CI; Confidence interval.

	Root Mean Standard Error	R2	Mean Absolute Error	95% CI ( *±* min)
Random Forest	1.04	0.92	0.8	1.17
BRNN	1.26	0.58	0.95	2.00

### Synthetic biotransformations approach for hypothesis generation

**Figure 4.**
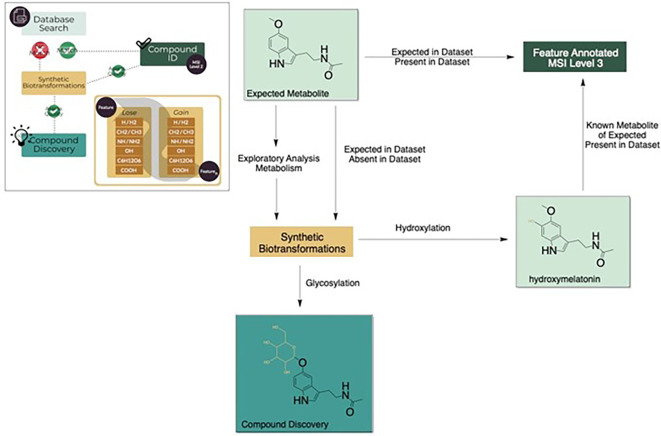
Overview of HormonomicsDB functionality.

One of the fundamental challenges which exists within the field of metabolomics is the scale of unknown unknowns within datasets. The average plant leaf has been estimated to contain >70,000 metabolites and with only a fraction of these having been characterized this leaves a vast chemical space for compound discovery as well as a significant challenge. ^10^ One advantage to living systems is that metabolites are generally not independent and are the result of enzymatic reactions, meaning there are a finite number of reactions that can occur. ^10^ While the vast number of potential novel metabolites is almost infinite, the number of basic reactions is more finite. In the synthetic biotransformations or logical algorithms approach developed in our group we use common chemical reactions such as (de) methylation, oxidation, reduction, (de) hydroxylation, (de) glycosylation, (de) carboxylation and apply these to a select subset of metabolites of interest to predict new pathways and metabolites in a sample based on a known starting point. ^4^^,^^6^^,^^11^^,^^10^ This approach uses the predicted change in monoisotopic mass of a specific metabolite by the addition or removal of a common moiety which can then be mined in the metabolomics dataset ( Figure 4).

**Figure 5.**
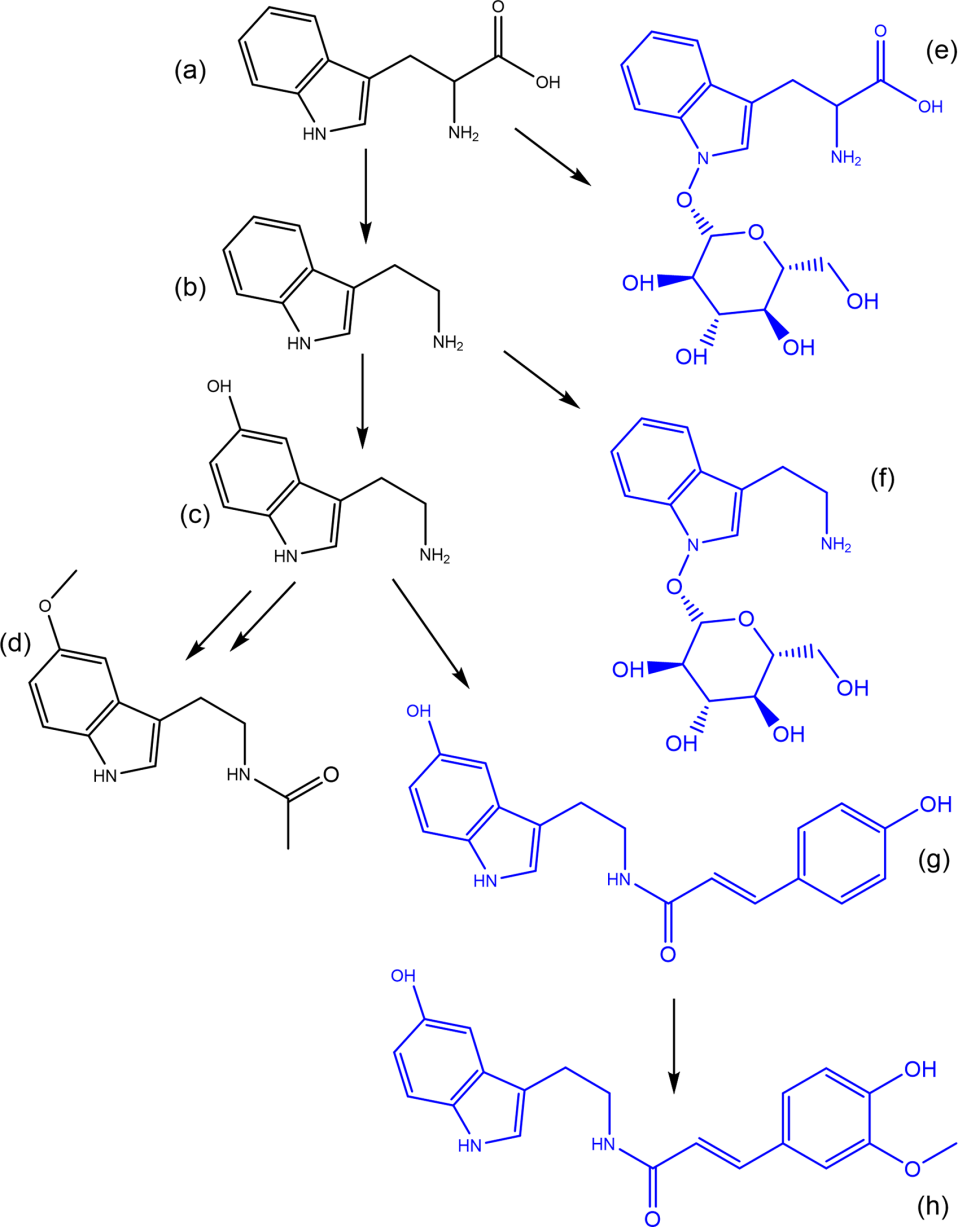
The pathway responsible for the biosynthesis of melatonin in plants starting from tryptophan (a) through to tryptamine (b) serotonin (c) and melatonin (d). The molecules in blue represent conjugates of these main metabolites in the pathway; (e) tryptophan-N-glucoside, (f) tryptamine-N-glucoside, (g) N-Coumaroyl serotonin, and (h) N-feruloylserotonin.

The synthetic biotransformations approach can also be used for the annotation of unknown knowns in a sample. This approach is particularly relevant for hormones as due to their biological activity at low concentrations, a common strategy is activation/deactivation of metabolites through simple reactions. It is possible that the active form is present at levels well below detection limits but a storage or deactivated form for transport is present at detectable levels and may still provide interesting biological context. Similarly, as the compounds are generally labile, a biologically active form of a hormone may be absent in a sample due to degradation during sample preparation or ionization but a modified form may be present. One of the best described examples of this is the classical phytohormone auxin which may be stored/deactivated/transported through methylation, glycosylation, and conjugation to amino acids among for example. ^8^^,^^22^^,^^23^ Phytohormones activity in some instances may also be enhanced or modified through conjugation or biotransformation as is the case for jasmonic acid, where the active form is jasmonic acid isoleucine, ^24^ or serotonin where phenolic conjugates such as feruloylserotonin are important in response to wounding, pathogen challenge and insect feeding. ^25^^,^^26^ A proof of concept of the application of synthetic biotransformations was performed on the pathway responsible for melatonin biosynthesis in plants using known conjugates ( Figure 5). In this proof of concept, two glycosylated metabolites are present, tryptophan-N-glycoside, and serotonin 5-O- *β*-glycoside. ^27^ These glycosylated forms may serve as inactive storage or transport forms of their parent molecule. Furthermore, the serotonin conjugates N-coumaroyl-serotonin and N-feruloyl-serotonin ( Figure 5, Table 6) have been found in several plant species where they serve important functions as defensive molecules. ^28^ There are potentially many more conjugates in this pathway that are yet to be identified, such as melatonin glycosides or N-coumaroyl-melatonin. The logic can also be performed in reverse, in the absence of a match for the parent compound melatonin the presence of for example while feruloyl-serotonin is not identified in the example cranberry dataset both a hydroxylated version and an aminated version are possible ( Table 6).

**Table 6.** Synthetic biotransformations for which the parent compound is absent in Brown *et al.* 2012 dataset but which indicate presence of the parent molecule. ^15^ Note that while the web-tool provides a predicted retention time this is for the parent compound not the transformed metabolite.

Compound name	Biotransformation	Actual m/z	Experimental m/z	Experimental RT	Predicted RT
7-hydroxy-ABA	M-OH+NH _3_	280.1549	280.1545	2.3782	5.58
7-hydroxy-ABA	M-OH+CH _3_	278.1518	278.161	5.7316	5.58
9-hydroxy-ABA	M-OH+CH _3_	278.1518	278.161	5.7316	5.49
Abscisic acid (ABA)	M-OH+CH _3_	262.1569	262.1596	3.6688	5.56
Abscisic acid (ABA)	M-OH+NH _3_	264.16	264.1576	2.478	5.56
Abscisic acid (ABA)	M-OH+CH _3_	262.1569	262.1458	3.8963	5.56
Abscisic acid (ABA)	M-OH+CH _3_	262.1569	262.1724	5.2207	5.56
9-hydroxy-ABA	M-OH+CH _3_	278.1518	278.1693	4.6964	5.49
7-hydroxy-ABA	M-OH+CH _3_	278.1518	278.1693	4.6964	5.58
Abscisic acid (ABA)	M-H+NH _2_	279.1471	279.1603	2.4673	5.56
2-methylthio-cis-zeatin riboside	M-H+2OH	430.1396	430.1473	5.12	4.13
Zeatin riboside-O-glucoside	M+CH _3_	528.2306	528.216	3.4882	4.05
2-methylthio-cis-zeatin	M-NH _2_+OH	266.0837	266.0744	5.4249	4.52
Melatonin (MEL)	M-CH _3_+OH	234.1004	234.1195	5.7546	4.88
Feruloyl serotonin	M+OH	369.145	369.1448	3.4826	3.83
Feruloyl serotonin	M-OH+NH _3_	352.1661	352.1854	3.9573	3.83
6-hydroxymelatonin	M-OH+NH _3_	248.1399	248.1329	2.4123	5.02
Melatonin (MEL)	M-H+OH	248.1161	248.1329	2.4123	4.88
Melatonin (MEL)	M.NH _2_	248.1399	248.1329	2.4123	4.88
Neophaseic acid	M-OH+NH _3_	280.1549	280.1545	2.3782	6.01
4-hydroxymelatonin	M-CH _3_+H	234.1004	234.1195	5.7546	4.97
N-sinapoyl Melatonin	M+CH _3_	453.2026	453.2179	5.3808	3.54
2-hydroxymelatonin	M-OH+CH _3_	248.1525	248.1329	2.4123	5.07
4-hydroxymelatonin	M-OH+NH _3_	248.1399	248.1329	2.4123	4.97
2-formylamino-benzaldehyde	M-OH+C _6_H _12_O _6_	284.1134	284.1037	5.8617	5.23
2,3-dihydroxyindole	M+C _6_H _12_O _6_	329.1111	329.1023	5.8638	4.98
N-Acetylserotonin	M.NH _2_	234.1243	234.1195	5.7546	4.63
N-Acetylserotonin	M-H+OH	234.1004	234.1195	5.7546	4.63
6-hydroxy-kynurenate	M-NH _2_+COOH	234.0164	233.9979	5.705	3.95
2,3-dihydroxyindole	M+C _6_H _12_O _6_	329.1111	329.1299	3.4602	4.98
2,3-dihydroxyindole	M+C _6_H _12_O _6_	329.1111	329.1299	6.098	4.98
2,3-dihydroxyindole	M+C _6_H _12_O _6_	329.1111	329.1163	4.876	4.98
2,3-dihydroxyindole	M+C _6_H _12_O _6_	329.1111	329.1212	5.5453	4.98

**Figure 6.**
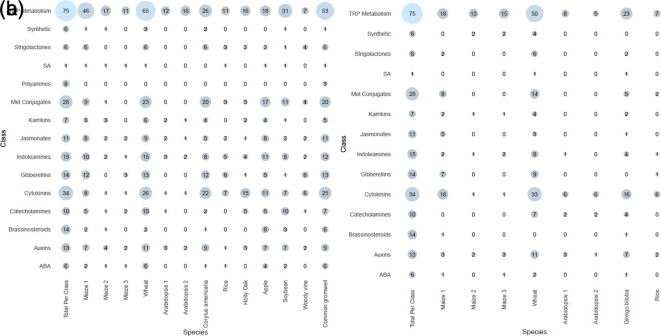
Number of compounds returned in each class for each of the species in the meta-analysis of data from Metabolomics Workbench, with the total number of compounds per class in HormonomicsDB given in the left most column. (a) Electrospray ionization (ESI) positive peak tables, and (b) ESI negative peak tables.

**Meta-analysis of previous studies** Tryptophan metabolism had the most hits of all the classes, across both ionization modes. This is not unexpected as tryptophan is involved in both primary and secondary metabolism, and tryptophan metabolites also make up the largest class in HormonomicsDB, with 75 tryptophan metabolites catalogued in HormonomicsDB ( Figure 6). Cytokinins and melatonin conjugates are the next two most abundant classes, respectively, however unlike tryptophan metabolism where the abundance is high across the species and experiments, these classes have a varying range in abundance across species. This demonstrates the sensitive nature of plant hormones, particularly with melatonin conjugates which are prone to degradation under light and oxidative conditions. ^18^

In species where there is an increase in tryptophan metabolism metabolites, an increase in melatonin conjugates and cytokinins is observed ( Figure 6). Increases in tryptophan have been observed to increase auxin and indolamine levels in *Hypericum perforatum* L. ^29^ Additionally, there is evidence suggesting auxin mediates indolamine biosynthesis in plants. ^29^ These mechanisms are not yet well understood and require further investigation. These empirical observations of increased tryptophan, auxin, and melatonin conjugate biosynthesis through the HormonomicsDB meta-analysis reveals the usefulness of the HormonomicsDB approach and how it could be applied to better understand how auxins interact with indolamine biosynthesis in plants.

Unlike the other -omics fields, particularly transcriptomics and genomics, variation between metabolomics experimental conditions, including extraction conditions, column chemistry, mobile phase composition, and ionization voltages makes meta-analyses challenging the data is not easily normalized and aligned (Supplementary File 2). ^30^ Our demonstration of a metabolomics meta-analysis through HormonomicsDB demonstrates the utility of having a standardized untargeted hormonomics protocol, which not only allows for the HormonomicsDB web-tool to be utilized for putative identification, but permits other researchers to analyze multiple metabolomics datasets without the need to account for variation between experiments.

## Conclusions

Phytohormones are an important class of metabolites as they regulate most physiological responses in plants, including reproduction, growth and development, stress response, and secondary metabolism. Our goal was to develop a tool that can putatively identify phytohormones from untargeted metabolomics datasets, as well as predicted phytohormone conjugates, to assist in the development of novel hypotheses about plant physiology. The putative identification of phytohormones from untargeted LC-MS metabolomics experiments provides valuable insight into plant physiology. We developed an easy to use and freely available webtool which allows users to mine their untargeted metabolomics data for phytohormones and potential conjugates. The web tool and accompanying standardized LC-MS protocol is unique in its specific focus on phytohormones and allows for compound identification up to MSI level 3 through incorporation of RT prediction.

The discovery of new hormone derived metabolites can generate novel hypotheses about plant physiology and signalling mechanisms. The synthetic biotransformations approach which is integrated into the tool could be applied to future studies to discover new bioactive secondary metabolites to explore how plants metabolize synthetic compounds including pesticides. ^4^^,^^10^ It is important to recognize that while the biotransformations approaches has significant power in annotation of unknowns it also has significant limitations and it should be used as a hypothesis generating tool rather than a confirmatory tool. For example, searching the same cranberry dataset ^15^ finds dozens of potential biotransformations which must be critically assessed for plausibility and feasibility, for example a compound which is not glycosylated cannot lose a sugar moiety. Additionally, as the biotransformations are predicted features, retention time prediction is not feasible for these compounds emphasizing the low confidence as a compound identification strategy alone. The ability to generate novel hypotheses for metabolite metabolism is also the strength of this approach. While some computational work and development is still necessary to implement this at larger scale, at the single metabolite or pathway level it can be a powerful approach to investigate and predict novel medicinal compounds, ^10^^,^^12^ understand metabolism of synthetic herbicides, ^6^ investigate regulation of morphogenesis ^4^^,^^31^ or microbiome interactions. ^32^ Additionally, our meta-analysis of 14 plant metabolomics experiments highlights the utility of using standardized metabolomics protocols, which allows for more comprehensive metabolomics meta-analyses.

Although this approach was designed to study phytohormones, there is significant overlap between phytohormones and endogenous human hormones. This approach can be used to explore small molecule hormones in animal samples as well, including in human tissues and bodily fluids. There is significant overlap between phytohormones and endogenous human hormones. As an example, serotonin, melatonin, and IAA are both phytohormones and endogenously produced hormones in humans. Aside from the role melatonin plays in regulating circadian rhythm in humans, it has been investigated for its role in reducing the impact that coronavirus disease 2019 (COVID-19) plays in supressing the damage caused by the virus SARS-CoV-2. ^33^^,^^34^ Serotonin metabolism is also altered in breast cancer cells leading to resistance in serotonin induced apoptosis and the serotonin conjugate, N-(p-coumaroyl) serotonin has been observed to induce apoptosis in breast cancer cells. ^35^^,^^36^ This highlights the multidisciplinary functionality of this approach to explore hormone biosynthesis in humans, plants and beyond.

## Author contributions

RTG: Conceptualization, Data curation, formal analysis, investigation, methodology, software, validation, visualization, writing original draft, writing review and editing, formal analysis; LAEE: Conceptualization, Data curation, visualization, writing original draft, writing review and editing, methodology; SJM: Conceptualization, data curation, funding acquisition, supervision, writing review and editing.

## Data and software availability

HormonomicsDB is free to use and accessible at HormonomicsDB.com. The code used to develop HormonomicsDB is available at https://github.com/plantSMART-UBC/HormonomicsDB. Formatting instructions, sample data, and output descriptions are available at HormonomicsDB.com and in the GitHub repository.

Borealis. Supporting information for “HormonomicsDB: A novel workflow for the untargeted analysis of plant growth regulators and hormones”. DOI: https://doi.org/10.5683/SP3/SIGTUN. ^37^

This project contains the following underlying data:

- Supporting information, consisting of a list of all phytohormones catalogued in HormonomicsDB and their accompanying chemical information (Supplementary File 1) and additional information regarding the studies assessed in the meta-analyses (Supplementary File 2).

### Extended data

Borealis. Supporting information for “HormonomicsDB: A novel workflow for the untargeted analysis of plant growth regulators and hormones”. DOI: https://doi.org/10.5683/SP3/SIGTUN. ^37^

- The PDF “Supporting Information for HormonomicsDB” contains background information discussing appropriate mass accuracy selection based on the mass detector used.

Data are available under the terms of the Creative Commons Zero “No rights reserved” data waiver (CC0 1.0 Public domain dedication).
