# Supplementary material for: HormonomicsDB: a novel workflow for the untargeted analysis of plant growth regulators and hormones
Source: F1000Res. 2024 Apr 8;11:1191. Originally published 2022 Oct 18. [Version 2] doi: 10.12688/f1000research.124194.2 (PMC11364965; doi:10.12688/f1000research.124194.2)
Supplement: Peer review history

# Reviewer report: Reviewer response for version 2

**DOI:** 10.5256/f1000research.161276.r264124

**Published:** 2024-08-30

**Authors:** Jian You Wang

**Affiliations:** 1 King Abdullah University of Science and Technology, Thuwal, Makkah Province, Saudi Arabia

The authors have addressed my comments in a rational way. Hope the improved MS version will come in the future.

Are the conclusions about the tool and its performance adequately supported by the findings presented in the article?

Yes

Is the rationale for developing the new software tool clearly explained?

Yes

Is the description of the software tool technically sound?

Partly

Are sufficient details of the code, methods and analysis (if applicable) provided to allow replication of the software development and its use by others?

Yes

Is sufficient information provided to allow interpretation of the expected output datasets and any results generated using the tool?

Partly

Reviewer Expertise:

Analytical biochemistry, Plant hormones, Strigolactones, Plant growth regulators

I confirm that I have read this submission and believe that I have an appropriate level of expertise to confirm that it is of an acceptable scientific standard.

# Reviewer report: Reviewer response for version 2

**DOI:** 10.5256/f1000research.161276.r264123

**Published:** 2024-05-13

**Authors:** R.J. Neil Emery, Ainsely Lewis

**Affiliations:** 1 Department of Biology, Trent University, Peterborough, ON, Canada; 2 Trent University, Peterborough, ON, Canada

The author has included all the suggestions.

Are the conclusions about the tool and its performance adequately supported by the findings presented in the article?

Yes

Is the rationale for developing the new software tool clearly explained?

Yes

Is the description of the software tool technically sound?

Yes

Are sufficient details of the code, methods and analysis (if applicable) provided to allow replication of the software development and its use by others?

Yes

Is sufficient information provided to allow interpretation of the expected output datasets and any results generated using the tool?

Yes

Reviewer Expertise:

Mass spectrometry-based metabolomics, phytohormone profiling, chemometrics

We confirm that we have read this submission and believe that we have an appropriate level of expertise to confirm that it is of an acceptable scientific standard.

# Reviewer report: Reviewer response for version 1

**DOI:** 10.5256/f1000research.136376.r174935

**Published:** 2023-06-12

**Authors:** Jian You Wang

**Affiliations:** 1 King Abdullah University of Science and Technology, Thuwal, Makkah Province, Saudi Arabia

HormonomicsDB is definitely required for the plant metabolomics field and it provides many advantages to identify compounds. While the paper is well written and clearly demonstrates how to use this platform, I have a few comments to, hopefully, improve the content.

1. What is the maximum mass tolerance (in Da and ppm) when identifying metabolites? Also, natural components usually contain isomers/isoforms, how to distinguish or interpret them by using HormonomicsDB? It would be necessary to guild users on how to transform Da to ppm.
2. Please include the explanations of the PGR Monoisotopic, PGR M+H, and so on to junior users.
3. It is a bit confusing between Table 4 and Table 5 in the m/z, what are the minimum decimal numbers of accurate mass (default setting) of particular metabolites in HormonomicsDB? In the above two cases, what was the mass tolerance (or acceptable error) to identify the compounds?
4. As a researcher in strigolactone (SL) biology, the methanol extraction would lead to the fast degradation of SLs. Could the author pay attention to this? In addition, the unarguable identification of a particular SL requires MS/MS (not only accurate mass). Therefore, if there would be MS/MS database in HormonomicsDB for advanced non-targeted metabolomics, it would be greatly helpful.
5. What is the minimum intensity (e3 or e4?) of peaks to be considered as metabolites instead of noise? 

Are the conclusions about the tool and its performance adequately supported by the findings presented in the article?

Yes

Is the rationale for developing the new software tool clearly explained?

Yes

Is the description of the software tool technically sound?

Partly

Are sufficient details of the code, methods and analysis (if applicable) provided to allow replication of the software development and its use by others?

Yes

Is sufficient information provided to allow interpretation of the expected output datasets and any results generated using the tool?

Partly

Reviewer Expertise:

Analytical biochemistry, Plant hormones, Strigolactones, Plant growth regulators

I confirm that I have read this submission and believe that I have an appropriate level of expertise to confirm that it is of an acceptable scientific standard, however I have significant reservations, as outlined above.

# Response

**Published:** 2024-01-10

**Authors:** Susan Murch

**Affiliations:** University of British Columbia, Canada

We thank reviewer 2 for reviewing our manuscript and for their appreciation of the need for untargeted metabolomics workflows for exploring phytohormones. Below, we have provided responses to each of the reviewer comments and how they were implemented to improve the manuscript.

**Comment #1:** *HormonomicsDB is definitely required for the plant metabolomics field and it provides many advantages to identify compounds. While the paper is well written and clearly demonstrates how to use this platform, I have a few comments to, hopefully, improve the content.*

**Author Response to Comment: **We appreciate that reviewer #2 has also identified the need for metabolomics approaches specifically tailored to the identification of phytohormones, specifically due to their labile and sensitive nature, as addressed in our manuscript.

**Comment #2:** *What is the maximum mass tolerance (in Da and ppm) when identifying metabolites? Also, natural components usually contain isomers/isoforms, how to distinguish or interpret them by using HormonomicsDB? It would be necessary to guild users on how to transform Da to ppm.*

**Author Response to Comment: **This was a comment also brought up by reviewer #1, and we have revised the manuscript to address this concern. Regarding mass tolerance, we updated HormonomicsDB to include an option to use ppm for mass tolerance. We have also added the following to the supplemental information:

 “Users can input their desired mass tolerance in Daltons (Da) or parts per million (ppm) in HormonomicsDB. The default mass tolerance is *± *0.01 Da; however, users can adjust this depending on the mass accuracy and resolution of their mass spectrometer. PPM is another common way to report mass tolerances, it is dependent on the value of the mass being measured. The relationship between Da and ppm is described by Equation 1.

 For example, a mass of 500 Da, the uncertainty at 1 ppm (calculated with Equation 1) is *±* 0.0005 Da, while the mass range for 100 Da is *± *0.0001 Da and *± *0.001 Da for a mass of 1000 Da.

 For untargeted LC-MS metabolomics workflows, it is strongly suggested that analysts use a mass detector with a high mass accuracy and resolving power, This improves the certainty in putative identification, by ensuring the mass measured is the correct mass and that ions with similar masses can be resolved from each other.

 Users of HormonomicsDB should consider the mass accuracy of their mass detector before selecting a mass tolerance. The mass accuracy ranges for the five most common mass spectrometers are given below ( https://fiehnlab.ucdavis.edu/projects/seven-golden-rules/accurate-mass).

**Supporting Table 1. **Mass accuracy ranges for the five most common mass spectrometers, given in parts per million (ppm).

**Type** **Mass Accuracy**

 FT-MS      0.1 - 1 ppm

 Orbitrap    0.5 - 1 ppm

 TOF-MS     3 - 5 ppm

 Q-TOF        3 - 5 ppm

 Triple Quad    3 - 5 ppm

 Regarding isomers/isoforms, it is often challenging in LC-MS to separate isomers as they can have relatively similar retention times. Additionally, MS/MS fragmentation often struggles to differentiate isomers. Ion mobility spectrometry – mass spectrometry (IMS-MS) is a relatively new technique which has been deployed to separate isomers in time based on their collisional cross section (CCS). This is an emerging technology and we expect that IMS will play an important role in future hormonomics studies. However, *in silico* CCS predictions are still in their infancy and IMS-MS capable instruments are still fairly inaccessible. Given these hurdles we cannot include IMS-MS in this current version of HormonomicsDB, however as these technologies become more accessible we will work toward including IMS-MS data in HormonomicsDB.

**Comment #3:** *Please include the explanations of the PGR Monoisotopic, PGR M+H, and so on to junior users.*

**Author Response to Comment: **To address any ambiguity to these terms we have added the following to the Methods section “Plant growth regulators”:

 “PGR Monoisotopic contains the monoisotopic mass for each phytohormone, the PGR M+H contains the m/z of each phytohormone as an M+H adduct, the PGR adducts contains the m/z of 7 common adducts (Table 1), and PGR biotransformations contains 27 common biotransformations (Table 1) as M+H adducts.”

**Comment #4:** *It is a bit confusing between Table 4 and Table 5 in the m/z, what are the minimum decimal numbers of accurate mass (default setting) of particular metabolites in HormonomicsDB? In the above two cases, what was the mass tolerance (or acceptable error) to identify the compounds?*

**Author Response to Comment: **We have updated table 4 and 6 to include more decimal points for both the experimental and library match m/z. Additionally, we have included the ppm difference, as HormonomicsDB now computes this. The data in Brown et al. was acquired on a TOF, therefore an uncertainty of 3-5 ppm is expected. We have added the following to the methods section discussing this:

 “The data in Brown *et al. *2012 was collected on a time of flight (TOF) mass spectrometer, which typically has a mass error of 3 to 5 ppm.”

**Comment #5:** *As a researcher in strigolactone (SL) biology, the methanol extraction would lead to the fast degradation of SLs. Could the author pay attention to this? In addition, the unarguable identification of a particular SL requires MS/MS (not only accurate mass). Therefore, if there would be MS/MS database in HormonomicsDB for advanced non-targeted metabolomics, it would be greatly helpful.*

**Author Response to Comment: **We understand that MS/MS is an important tool for discovering and identifying phytohormones in untargeted studies, however, we found that current tools for *in silico *prediction of low energy MS/MS spectra often provide incorrect predictions, we felt it would be inappropriate to add MS/MS to HormonomicsDB in this publication. But, as *in silico *MS/MS prediction tools improve and more phytohormone standards become accessible, we hope to release another version of HormonomicsDB with MS/MS searching capabilities.

**Comment #5:** *What is the minimum intensity (e3 or e4?) of peaks to be considered as metabolites instead of noise?*

**Author Response to Comment: **Since HormonomicsDB takes aligned peak tables, the signal to noise (S/N) cut-off would have been determined during the alignment process, as this is the most common approach vendor software currently use for selecting chemical features prior to alignment. Additionally, some software outputs the aligned peak table with relative intensities. To address this concern we added the following to the Methods: “HormonomicsDB makes no assumptions about signal to noise (S/N) or other sensitivity figures of merit as these are typically made during this peak alignment step.”

# Reviewer report: Reviewer response for version 1

**DOI:** 10.5256/f1000research.136376.r153643

**Published:** 2022-11-08

**Authors:** R.J. Neil Emery, Ainsely Lewis

**Affiliations:** 1 Trent University, Peterborough, ON, Canada; 2 Department of Biology, Trent University, Peterborough, ON, Canada

**Review by Ainsely Lewis, Ph.D., and Professor RJ Neil Emery**

**Good points/Strengths of article: **

1. HormonomicsDB undoubtedly is a needed comprehensive tool to screen metabolites/phytohormones and plant growth regulators putatively. This tool is no doubt a novel tool that will aid researchers using mass spectrometry-based metabolomics for hormones in either plant or animal systems.
2. Making choices of what classes or types of compounds to search for is particularly difficult. Therefore, this is a particularly helpful tool for narrowing metabolomics studies into a search for signaling molecules. It offers a focused study on a higher level in that it can uncover important compounds that have wider reaching or pleiotropic effects.
3. This is especially good for screening for these hormones especially if they are not known to be present in a given system of study.
4. Knowing putative compounds in an extraction procedure with various factors (extraction buffer used, solvents, using SPE or not), can really aid in targeted analysis, or to help develop a method to maximize extraction of hormones/phytohormones of interest.
5. This manuscript is quite an undertaking! This is a well written and well-done manuscript, that is scientifically sound.
6. Introduction was well done and nicely sets the pace for readers. Methodology was thorough and straightforward. Results and discussion section was thorough. Liked the COVID angle in the conclusions. The grammar and word choices used in the manuscript was good as well.

**Approved with reservations. Minor adjustments/revisions needed.**

**Minor suggestions - Things for clarification for other users/readers with commentary.**

 These are suggestions that we believe can aid in improving the manuscript:

1. Not everyone is aware of the relationship with Dalton (Da) and parts per million (ppm). This is speaking about mass tolerance, especially looking on m/z values near the theoretical values of phytohormones/plant growth regulators. Conversion from Da to ppm is dependent on the m/z examined as you are aware. I think it is important for users to be aware of this, as not everyone may have an intense Chemistry background. We suggest that either adding this in the text (supplemental or otherwise) would really aid the manuscript and users new to mass spectrometry-based metabolomics. Additionally, you could mention that for the data that is downloaded from HormonomicsDB, that to know the ppm error used, that they use the formula: observed mass – theoretical mass/theoretical mass * 10 ^^6^ to know what error in ppm. The default for HormonomicsDB is ±0.01 Da. How much Daltons error wise do you recommend people using? In other words, if you have an ion mass of 1000.1356, and you want a desired ppm error of 5, how much would it be in daltons, or how much deviation can you expect? Using this formula (i.e., deviation in daltons with a given ion mass), especially, would clear up some ambiguity using either ppm or daltons, as well as to guide users/readers.
2. We suggest that you should mention that the acceptable error limits that would be dependent on the mass spectrometer of question. For example, using 20 ppm on a high-resolution mass spectrometry instrument especially with MS1 data is not kindly looked on some individuals in the mass spectrometry world. For example, 5-10 ppm error or less for Orbitrap is acceptable. This is not necessarily needed in the main text, but can be put in the supplemental, as this is not the main message of the manuscript. This was mentioned as studies you screened are from the Orbitrap and Q-TOF instruments.
3. Having being users of HormonomicsDB, we think it is important to mention on the HormonomicsDB website the LC gradient that was used for the study. You mention it in the manuscript, which is good, but having it on the website somewhere will let the user be reminded that the LC gradient used in the paper may not be like theirs. Too much reliance on RTs may result in false negatives or positives. Even among different labs, the same machine, same columns, and mobile phase gradients can result in significantly different Rt’s. The readers should be more in tune to the elution points of compounds relative to others or external standards.
4. Although the note about RT match is dependent on machine learning (which is a nice and novel approach by the way), we suggest that the authors should emphasize more to the reader that they should stick with exact mass (m/z) within a certain error margin, should they use another LC gradient, with various run times (solvents, columns, etc).
5. In the supplemental information, especially supplemental file 1, although for the table of phytohormones M+H was used, we suggest that the masses from the [M-H] ^-^ ionization mode should be added as well. We also suggest that the synthetic compounds should not be grouped with the plant growth regulators or their precursor compounds and/or intermediates. Rather, separate them into different sheets. For example: predicted metabolites (Table 1), training standards (Table 2), and synthetic compounds (Table 3). Many people won’t care about glyphosate or thidiazuron as much as other compounds that are endogenous such as Spermidine, Indoleacetate (IAA), or Gibberellins (GAs). While it is still good to know if glyphosate was used in the plant organism of interest, for the supplemental, maybe put glyphosate and other synthetic compounds to themselves. That way, the focus can be on phytohormones/plant growth regulators and other related compounds from Tryptophan metabolism. If the readers want, they can consider the other synthetic ones just to see. Putting them in different tables also helps in reading.
6. We tested HormonomicsDB with XCMS Online results from untargeted metabolomics data and it (i.e., HormonomicsDB) works! Targeting Gibberellins via extraction can be a challenge, but they were putatively identified in negative mode data extracted using XCMS Online! I think it would aid the manuscript to mention that XCMS Online data can be formatted for use in HormonomicsDB! It gives surprising results! Although XCMS Online is a tool used for aligning peaks/mass features, we still must check the actual retention time in the processing software that comes with the mass spectrometer to make sure that the peak is a good one. It is wonderful that you mentioned MetaboAnalyst but think about XCMS Online or other peak/feature aligning methods as well. XCMS Online and MetaboAnalyst can be used in tandem to make meaningful information. One that readers can try is a recent peak/feature aligning server/software: Metaboseek (published this year). You did mention Misra *et al*. 2021’s comprehensive review! We suggest that you should mention other open-source tools that can be used to preprocess data that could be used with HormonomicsDB. It would bolster the strength of HormonomicsDB as being more versatile than just a standalone tool.
7. While not the pivot of the study, the custom database output does not get enough credit in this paper. We tried primary metabolites with XCMS Online data and found compounds based on m/z values! We did not consider the RT as a factor as our lab method is 1/3 ^rd^ the length of the lab method mentioned in this manuscript. *But this goes to show that users can use a subset of hormones and other key metabolites they are only interested in to get an output, for more targeted analyses later.* We believe that this is another strength of using HormonomicsDB custom database that should be emphasized more.
8. When do you use biotransformations? In using the software, it can be confusing. Can you clarify this part a bit?
9. Letting the user know what the ups and downs or advantages and disadvantages of each function (i.e., PGR monoisotopic, PGR M-H or M+H, PGR Adducts, PGR Biotransformations) would be helpful. What have you found using these? Some commentary about how to use these functions alone, or in support of each other would be quite helpful.

Are the conclusions about the tool and its performance adequately supported by the findings presented in the article?

Yes

Is the rationale for developing the new software tool clearly explained?

Yes

Is the description of the software tool technically sound?

Yes

Are sufficient details of the code, methods and analysis (if applicable) provided to allow replication of the software development and its use by others?

Yes

Is sufficient information provided to allow interpretation of the expected output datasets and any results generated using the tool?

Yes

Reviewer Expertise:

Mass spectrometry-based metabolomics, phytohormone profiling, chemometrics

We confirm that we have read this submission and believe that we have an appropriate level of expertise to confirm that it is of an acceptable scientific standard, however we have significant reservations, as outlined above.

# Response

**Published:** 2024-01-10

**Authors:** Susan Murch

**Affiliations:** University of British Columbia, Canada

We thank reviewer 1 for pointing out what they identified as strengths within our article and ultimately in our novel metabolomics tool/workflow. We appreciate their helpful feedback and comments which we believe will strengthen our manuscript and metabolomics tool. Below we have provided responses to each of the reviewer comments and how they were implemented to improve the manuscript.

**Comment #1:** Not everyone is aware of the relationship with Dalton (Da) and parts per million (ppm). This is speaking about mass tolerance, especially looking on m/z values near the theoretical values of phytohormones/plant growth regulators. Conversion from Da to ppm is dependent on the m/z examined as you are aware. I think it is important for users to be aware of this, as not everyone may have an intense Chemistry background. We suggest that either adding this in the text (supplemental or otherwise) would really aid the manuscript and users new to mass spectrometry-based metabolomics. Additionally, you could mention that for the data that is downloaded from HormonomicsDB, that to know the ppm error used, that they use the formula: observed mass – theoretical mass/theoretical mass * 10^6 to know what error in ppm. The default for HormonomicsDB is ±0.01 Da. How much Daltons error wise do you recommend people using? In other words, if you have an ion mass of 1000.1356, and you want a desired ppm error of 5, how much would it be in daltons, or how much deviation can you expect? Using this formula (i.e., deviation in daltons with a given ion mass), especially, would clear up some ambiguity using either ppm or daltons, as well as to guide users/readers.

**Comment #2:** *We suggest that you should mention that the acceptable error limits that would be dependent on the mass spectrometer of question. For example, using 20 ppm on a highresolution mass spectrometry instrument especially with MS1 data is not kindly looked on some individuals in the mass spectrometry world. For example, 5-10 ppm error or less for Orbitrap is acceptable. This is not necessarily needed in the main text, but can be put in the supplemental, as this is not the main message of the manuscript. This was mentioned as studies you screened are from the Orbitrap and Q-TOF instruments.*

**Author Response to Comment: **We have chosen to address comment #1 and #2 together since they are closely related. We have added a Supplemental Information (SI) file to address this issue regarding mass tolerance in Da and ppm. We have added the following to the SI under the heading “Selecting Mass Tolerance”. Reference has been made to this section in the SI on page 4 in the manuscript under the section “Data inputs and outputs”: We have also updated HormonomicsDB to include an option to use ppm for mass tolerance.

 “Users can input their desired mass tolerance in Daltons (Da) or parts per million (ppm) in HormonomicsDB. The default mass tolerance is *± *0.01 Da; however, users can adjust this depending on the mass accuracy and resolution of their mass spectrometer. PPM is another common way to report mass tolerances, it is dependent on the value of the mass being measured. The relationship between Da and ppm is described by Equation 1.

 For example, a mass of 500 Da, the uncertainty at 1 ppm (calculated with Equation 1) is *±* 0.0005 Da, while the mass range for 100 Da is *± *0.0001 Da and *± *0.001 Da for a mass of 1000 Da.

 For untargeted LC-MS metabolomics workflows, it is strongly suggested that analysts use a mass detector with a high mass accuracy and resolving power, This improves the certainty in putative identification, by ensuring the mass measured is the correct mass and that ions with similar masses can be resolved from each other.

 Users of HormonomicsDB should consider the mass accuracy of their mass detector before selecting a mass tolerance. The mass accuracy ranges for the five most common mass spectrometers are given below ( https://fiehnlab.ucdavis.edu/projects/seven-golden-rules/accurate-mass).

**Supporting Table 1. **Mass accuracy ranges for the five most common mass spectrometers, given in parts per million (ppm).

**Type** **Mass Accuracy**

 FT-MS             0.1 - 1 ppm

 Orbitrap           0.5 - 1 ppm

 TOF-MS              3 - 5 ppm

 Q-TOF                3 - 5 ppm

 Triple Quad         3 - 5 ppm

**Comment #3:** *Having being users of HormonomicsDB, we think it is important to mention on the HormonomicsDB website the LC gradient that was used for the study. You mention it in the manuscript, which is good, but having it on the website somewhere will let the user be reminded that the LC gradient used in the paper may not be like theirs. Too much reliance on RTs may result in false negatives or positives. Even among different labs, the same machine, same columns, and mobile phase gradients can result in significantly different Rt’s. The readers should be more in tune to the elution points of compounds relative to others or external standards.*

**Comment #4:** *Although the note about RT match is dependent on machine learning (which is a nice and novel approach by the way), we suggest that the authors should emphasize more to the reader that they should stick with exact mass (m/z) within a certain error margin, should they use another LC gradient, with various run times (solvents, columns, etc).*

**Author Response to Comment: **We are going to address comments #3 and #4 together as they discuss similar concerns. We will add a mention of the RT and gradient used on the landing page for HormonomicsDB. Additionally, we will add a link to this manuscript there so users can refer to the paper before using the tool. We feel that this should address the concerns in comment #3. We have added the following statement to the manuscript to the section “Retention time prediction” in the Result and discussion:

 “While RT predictions help to increase confidence in putative identification, it is important to note that differences in gradient, column, or solvents can impact the RT of analytes. The users should place more emphasis on the accurate mass matching and use RT prediction results to confirm if the elution pattern or order is as expected and elution of the analyte occurs at a reasonable time within the gradient”.

**Comment #5:** *In the supplemental information, especially supplemental file 1, although for the table of phytohormones M+H was used, we suggest that the masses from the [M-H]- ionization mode should be added as well. We also suggest that the synthetic compounds should not be grouped with the plant growth regulators or their precursor compounds and/or intermediates. Rather, separate them into different sheets. For example: predicted metabolites (Table 1), training standards (Table 2), and synthetic compounds (Table 3). Many people won’t care about glyphosate or thidiazuron as much as other compounds that are endogenous such as Spermidine, Indoleacetate (IAA), or Gibberellins (GAs). While it is still good to know if glyphosate was used in the plant organism of interest, for the supplemental, maybe put glyphosate and other synthetic compounds to themselves. That way, the focus can be on phytohormones/plant growth regulators and other related compounds from Tryptophan metabolism. If the readers want, they can consider the other synthetic ones just to see. Putting them in different tables also helps in reading.*

**Author Response to Comment:** We appreciate these great suggestions for the database content. The synthetic compounds provide some useful and comparatively inexpensive standards to train the chromatography model and are useful for the analytical method but not necessarily the biological question. We are working on software updates will incorporate a tool to distinguish between synthetics and naturally occurring compounds in a future version.

**Comment #6:** *We tested HormonomicsDB with XCMS Online results from untargeted metabolomics data and it (i.e., HormonomicsDB) works! Targeting Gibberellins via extraction can be a challenge, but they were putatively identified in negative mode data extracted using XCMS Online! I think it would aid the manuscript to mention that XCMS Online data can be formatted for use in HormonomicsDB! It gives surprising results! Although XCMS Online is a tool used for aligning peaks/mass features, we still must check the actual retention time in the processing software that comes with the mass spectrometer to make sure that the peak is a good one. It is wonderful that you mentioned MetaboAnalyst but think about XCMS Online or other peak/feature aligning methods as well. XCMS Online and MetaboAnalyst can be used in tandem to make meaningful information. One that readers can try is a recent peak/feature aligning server/software: Metaboseek (published this year). You did mention Misra et al. 2021’s comprehensive review! We suggest that you should mention other opensource tools that can be used to preprocess data that could be used with HormonomicsDB. It would bolster the strength of HormonomicsDB as being more versatile than just a standalone tool.*

**Author Response to Comment: **This is an important point that is often understated in the literature, since many analysts do not consider how raw data files are converted into a peak table, or the “X” block for putative ID or statistical analysis. To address this, we added the following to the section “Data input and output”:

 “Users can generate peak tables for upload to HormonomicsDB a number of ways, including with vendor software, or open source packages. These software take raw data files collected from individual chromatographic runs, and align the peaks to generate a single peak table which describes all the samples in the metabolomics experiment. These open source tools include XCMS (xcmsonline.scripps.edu), MetaboAnalyst (metaboanalyst.com), mzMine (mzmine.github.io), and Metaboseek (metaboseek.com).”

**Comment #7:** *While not the pivot of the study, the custom database output does not get enough credit in this paper. We tried primary metabolites with XCMS Online data and found compounds based on m/z values! We did not consider the RT as a factor as our lab method is 1/3rd the length of the lab method mentioned in this manuscript. But this goes to show that users can use a subset of hormones and other key metabolites they are only interested in to get an output, for more targeted analyses later. We believe that this is another strength of using HormonomicsDB custom database that should be emphasized more.*

**Author Response to Comment: **To emphasize the utility of the HormonomicsDB custom search function we have changed this section from a sub section to its own section in the manuscript (in the methods), and discussed the utility of this function by adding the following to the Results and discussion section “HormonomicsDB web-tool functionality and interface”:

 “Additionally, the “HormonomicsDB custom search” function is a novel feature within HormonomicsDB which allows users to search their peak table against their own database of metabolites. This is the first such report of a feature that allows users to queue with their own database. We hope to continue growing this function to allow users to queue against other databases by accessing them through an application programming interface (API) in the HormonomicsDB environment.”

**Comment #8:** *When do you use biotransformations? In using the software, it can be confusing. Can you clarify this part a bit?*

**Comment #9:** *Letting the user know what the ups and downs or advantages and disadvantages of each function (i.e., PGR monoisotopic, PGR M-H or M+H, PGR Adducts, PGR Biotransformations) would be helpful. What have you found using these? Some commentary about how to use these functions alone, or in support of each other would be quite helpful.*

**Author Response to Comment: **Comments #8 and #9 are related, so we will address them together. We added the following to the end of the section in methods “Data input and output”:

 “The databases queued against depend on the structure of the input data and the users hypothesis. Certain software, particularly for Fourier transform mass spectrometry (FT-MS) data, convert ion masses to monoisotopic mass before exporting a peak table. If this is the case, the user can queue against the “PGR Monoisotopic” database.

 In ESI-LC-MS, the most common adduct is M+H in ESI positive mode and M-H in ESI negative mode. To queue for molecular ions only in the untargeted dataset, users should select the “PGR M+H” or “PGR M-H” databases, depending on their ionization mode. Adducts are often encountered in ESI-LC-MS, especially in ESI positive mode, as metal ions tend to form cations in aqueous solution. If the intensity for a particular PGR is decreased, or the PGR is absent when searching for molecular ion, the user can search for the common adducts of the PGRs in HormonomicsDB by queueing against the “PGR Adducts” database. Searching for the molecular ion and adducts at the same time is permissible in HormonomicsDB and allows the user to detect PGRs present in both forms.

 Biotransformations allow the user to search for modified forms of PGRs in their metabolomics dataset. This is useful when the goal is to identify conjugates and metabolites of PGRs in plants. The “PGR Biotransformations” database can be searched concurrently with the “PGR M+H” database, to identify PGRs in their native state and as conjugates. This approach can lead to the generation of new hypotheses in plant physiology and metabolism.”
